# A case of laryngopharyngeal reflux‐associated chronic cough: Misinterpretation of treatment efficacy causes diagnostic delay

**DOI:** 10.1002/jgf2.348

**Published:** 2020-07-18

**Authors:** Asuka Kikuchi, Ryuichi Kawamoto, Junki Mizumoto, Taichi Akase, Daisuke Ninomiya, Teru Kumagi

**Affiliations:** ^1^ Department of Community Medicine Graduate School of Medicine Ehime University Ehime Japan; ^2^ Department of Family Practice Ehime Seikyou Hospital Ehime Japan

**Keywords:** chronic cough, laryngopharyngeal reflux, mosapride, proton‐pump inhibitor, silent aspiration

## Abstract

A 62‐year‐old woman presented with a dry cough lasting 18 months. She had previously been examined by multiple doctors, but no abnormalities were observed. Several medications such as rabeprazole and inhaled corticosteroids were administered as test treatments without any improvement. Therefore, the possibility of biological disease, including acid reflux, had been mistakenly ruled out. We examined the sputum gram stain. The result showed phagocyted normal bacterial flora, suggesting aspiration. Laryngoscopy revealed edema of the arytenoid cartilage. The patient was finally diagnosed with laryngopharyngeal reflux and silent aspiration. This case suggested that the ineffectiveness of proton‐pump inhibitors cannot always exclude the presence of reflux disease and the usefulness of gram stain examination to detect silent aspiration.

## Graphical abstract

A 62‐year‐old woman presented with a dry cough lasting 18 months. We proceeded the sputum gram stain and laryngoscopy. The result showed phagocyted normal bacterial flora, suggesting aspiration and edema of the arytenoid cartilage by acid. The patient was finally diagnosed with laryngopharyngeal reflux.

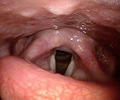

## 1 INTRODUCTION

Laryngopharyngeal reflux (LPR) is defined as the reflux of acid and enzymes, such as pepsin, into the pharyngolarynx. Although gastroesophageal reflux disease (GERD) is also acid reflux disease, there are some differences between them. Firstly, the dysfunction location is different between GERD and LPR. The lower esophageal sphincter dysfunction is the main cause of GERD, and the upper sphincter dysfunction is the main cause of LPR.^1^ Secondly, the sensitivity of epithelium to acid is also different. While the epithelium of the esophagus expresses varieties of carbonic anhydrases (CAs), which protect the epithelium from acid reflux, the epithelium of laryngopharynx expresses less types of CAs, which result in vulnerability to acid.^2^ This histological difference can explain why LPR patient does not always have esophagitis.^2^, ^3^ LPR is one of the most common causes of chronic coughs.^3^ LPR usually poses a diagnostic challenge for physicians because there is no consensus on LPR diagnosis; not all LPR patients show abnormality in laryngoscopy, and 24‐hour pH testing may miss the minute acid exposure.^4^ Herein, we report a case of chronic cough caused by LPR and aspiration whose diagnostic clincher was the identification of phagocyted normal bacterial flora in the patient sputum.

## 2 CASE PRESENTATION

A 62‐year‐old woman was referred to our facility with a persistent dry cough lasting for 18 months. The patient had never smoked. When the dry cough developed, she saw her family physician and was prescribed dihydrocodeine. Her symptoms did not improve, and she took the following medications sequentially: clarithromycin, pranlukast, and one‐month rabeprazole, all of which did not alleviate her cough. She was referred to an otolaryngologist, and it was determined that rhinitis or sinusitis was unlikely. Four months after the onset, she was referred to another hospital, and a pulmonologist performed laboratory examinations, spirometry, an alveolar nitric oxide concentration test, and computer tomography scan (CT scan) to rule out lung cancer; chronic infectious disease, including tuberculosis; and interstitial pneumonitis. These examinations revealed no particular abnormalities. The pulmonologist tried an inhaled corticosteroid and tiotropium bromide as test treatments. However, neither of them worked well. The patient was tentatively diagnosed with psychogenic cough. Her symptoms worsened, and she was referred to our hospital.

**FIGURE 1:**
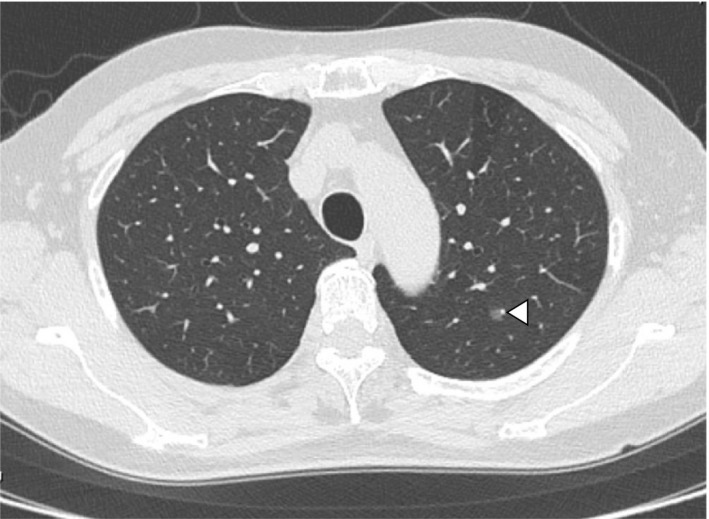
A CT image of the chest. An arrowhead indicates ground‐glass attenuation in the left lower lobe

**FIGURE 2:**
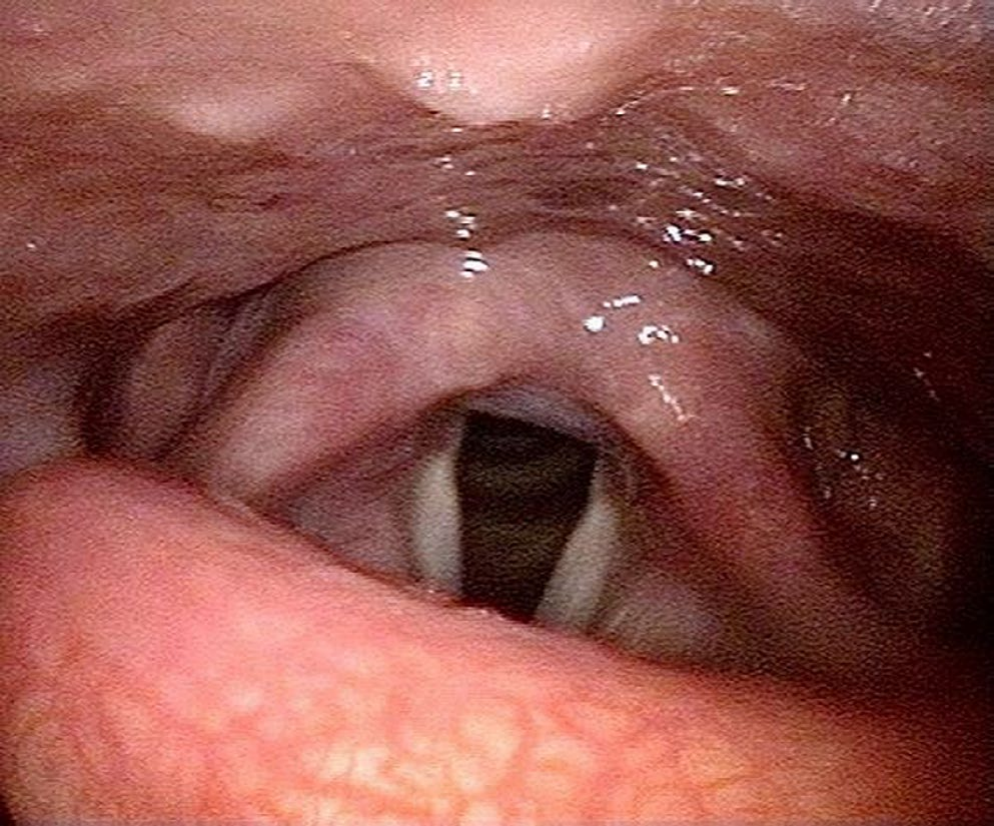
Edema of arytenoid cartilage revealed by laryngoscopy

The patient had no previous contact with sick people or animals, and she had never traveled abroad. The symptoms did not show a diurnal variation and were not related to menstruation, body position, nor environmental factors. She reported no symptoms other than the cough. On physical examination, she was afebrile and appeared well with normal vital signs. Facial knock pain was absent. No ulceration was found in her oropharynx. There were no palpable cervical or supraclavicular lymph nodes. Cardiac examination showed no abnormalities, and her breath sound was clear with no rales. Her legs and feet were not swollen. There was no rash on her skin. No neurological abnormalities were shown. The laboratory examination did not reveal any particular findings. The repeated chest CT scan revealed ground‐glass attenuation in the left lower lobe, which we think has no relation to her symptom (Figure 1), and no other abnormalities were found. Even though her cough was nonproductive, we examined the sputum culture test by induction with nebulized 5% saline. The result showed normal phagocyted normal bacterial flora, which suggested chronic aspiration. Laryngoscopy revealed edema of the arytenoid cartilage and slight epiglottic inversion dysfunction because of its malformation, which seemed to be congenital (Figure 2). We ordered a brain MRI to rule out the possibility of bulbar paralysis and stroke and revealed no abnormalities. Considering these results, we gave the final diagnosis: chronic cough caused by slight epiglottic inversion dysfunction because of malformation, silent aspiration because of aging, and LPR. We prescribed vonoprazan and mosapride, expecting the clearance of gastric acid. Symptoms gradually disappeared within two months, and finally, the patient was relieved from chronic cough for the first time in the two years after onset.

## 3 DISCUSSION

Chronic cough is one of the most common chief complaints in primary care settings. It is challenging for physicians to diagnose chronic cough. In this case, a persistent cough was caused by LPR in combination with silent aspiration and epiglottic inversion dysfunction. LPR was probably the main cause because treatment with vonoprazan and mosapride reduced her symptoms. It is also possible that silent aspiration because of aging and epiglottic inversion dysfunction may exacerbate the symptoms of chronic cough.

While LPR is one of the most common causes of chronic cough, the underlying mechanism and treatment are not sufficiently understood in Japan.^5^ The following facts are not adequately apparent among Japanese physicians: LPR patients do not always have detectable inspection abnormalities and proton‐pump inhibitors (PPIs) may not work well on LPR patients without esophageal mucosal lesions.^1^, ^6^, ^7^, ^8^ Therefore, LPR patients without detectable abnormalities and poor response to PPIs may be misdiagnosed. In our case, the possibility of LPR had been mistakenly ruled out by several physicians because of the ineffectiveness of treatment of PPIs, which resulted in diagnostic delay. The gram stain of her sputum, however, implied the possibility of aspiration, which motivated us to proceed with laryngoscopy. Edema of the arytenoid cartilage and slight epiglottic inversion dysfunction because of malformation were revealed by laryngoscopy, resulting in a diagnosis.

Initial treatment of LPR is dietary and behavioral modification.^6^, ^7^ Chronic cough related to LPR is caused by these two mechanisms: the direct stimulation of the laryngopharyngeal mucosa by acid and enzymes and vagal reflex evoked by reflux into the esophagus and laryngopharyngitis.^9^ Therefore, avoiding acidic foods and exercises that increase intra‐abdominal pressure is important. Some researchers suggest that the combined use of gastric acid clearance stimulants such as itopride or Rikkunshito, an herbal medicine, to PPIs, can ease coughs and irritable sensations caused by LPR.^10^, ^11^ We chose a combination of vonoprazan and mosapride which belongs to the same class as itopride. This treatment worked well.

In this case, physicians’ over‐reliance on PPIs leads to the diagnostic delay. Empirical PPI use is common as test treatment for chronic cough because of reflux diseases such as GERD. However, the diagnostic value of the PPI test for the detection of nonerosive reflux disease is reported to be 67%, which is not that high.^12^ Our case suggests that the ineffectiveness of PPIs cannot always exclude the presence of acid reflux disease. Primary care physicians who refer patients to specialists should be aware of this pitfall.

## 4 CONCLUSION

We report a case of chronic cough caused by LPR in combination with silent aspiration and epiglottic inversion dysfunction. When diagnosing chronic cough in a primary care setting, a sputum gram stain may be useful to detect aspiration even if cough is dry. To avoid the diagnostic delay, physicians should be aware of the diagnostic values of treatment and examination.

## CONFLICT OF INTEREST

The authors have stated explicitly that there are no conflicts of interest in connection with this article.

## ETHICAL APPROVAL

The informed consent was obtained to publish this case report.
